# Genome-wide long non-coding RNA expression profile and its regulatory role in the ileocecal valve from *Mycobacterium avium* subsp. *paratuberculosis*-infected cattle

**DOI:** 10.3389/fvets.2025.1601267

**Published:** 2025-06-05

**Authors:** Gerard Badia-Bringué, Victoria Asselstine, Ángela Cánovas, Marta Alonso-Hearn

**Affiliations:** ^1^Department of Animal Health, NEIKER-Basque Institute of Agricultural Research and Development, Basque Research and Technology Alliance (BRTA), Derio, Spain; ^2^Department of Animal Biosciences, Center for Genetic Improvement of Livestock, University of Guelph, Guelph, ON, Canada

**Keywords:** long non-coding RNAs, paratuberculosis, transcriptomics, RNA-Seq, biomarkers, gene regulation

## Abstract

Bovine paratuberculosis (PTB) is a chronic enteritis caused by *Mycobacterium avium* subsp. *paratuberculosis* (MAP), which results in significant economic losses to the dairy industry worldwide. Long non-coding RNAs (lncRNAs) play a crucial role in regulating the host immune response due to their interaction with transcripts in proximity. However, their annotation in cattle remains limited, and their role in cattle naturally infected with MAP has not been fully explored. In this study, lncRNAs were identified in the transcriptome of ileocecal valve samples from control cows without lesions (*N* = 4) and with PTB-associated focal (*N* = 5) and diffuse (*N* = 5) lesions in intestinal tissues using RNA sequencing. The raw reads were uploaded into the CLC Bio Genomics Workbench, and the trimmed reads were mapped to the *Bos taurus* ARS_UCD1.2.109 reference genome using the Large Gap Read Mapping tool. The resulting annotation allowed the identification of 1,434 LncRNAs, 899 of which were novel, using the FlExible Extraction of LncRNA pipeline. LncRNA differential expression (DE) analysis performed with *DESeq2* allowed the identification of 1, 6, and 2 DE lncRNAs in the comparisons of cows with focal lesions *versus* (*vs*) controls, diffuse lesions *vs.* controls, and diffuse *vs.* focal lesions, respectively. Best lncRNA partner analysis identified expression correlations between the *lncRNA1086.1*, lncRNA *ENSBTAG00000050406*, and *lncRNA_2340.1*, and the *Inactive Phosphatidylinositol 3-Phosphatase 9* (*MTMR9*), *GM Domain Family member B (RGMB),* and the *homeobox A6* (*HOXA6*), respectively. The *MTMR9* negatively regulates apoptosis, the *RGMB* positively regulates IL-6 expression, and the *HOXA6* regulates cell differentiation and inflammation. The results of the quantitative trait locus (QTL) enrichment analysis showed that the DE lncRNAs were located in genomic regions previously associated with clinical mastitis, HDL cholesterol, bovine tuberculosis, paratuberculosis, and bovine leukosis susceptibility. The identified DE lncRNAs could allow the development of novel PTB diagnostic tools and have potential applications in breeding strategies for PTB-resistant cattle.

## 1 Introduction

Bovine paratuberculosis (PTB), also known as Johne’s disease, is a chronic granulomatous enteritis that affects ruminants worldwide. The disease is caused by *Mycobacterium avium* subsp. *paratuberculosis* (MAP) and is primarily characterized by reduced milk production and weight loss. Global economic losses due to PTB are estimated to exceed $1.5 billion annually, with $198.42 million in the United States and $364.31 million in Europe (1, 2). These losses are mainly due to decreased milk production, decreased pregnancy rates, increased management costs, and premature culling of infected animals. Indeed, bovine PTB is considered endemic in the United States and Europe, with more than 50% of herds testing positive for anti-MAP antibodies by ELISA (3). Furthermore, scientific evidence links MAP to human inflammatory bowel disease (IBD), Crohn’s disease, autoimmune diseases, colorectal cancer, and Alzheimer’s disease (4–6). This potential threat to human health has increased interest in PTB and the development of more sensitive diagnostic and control methods.

Transmission of MAP typically occurs early in an animal’s life through the ingestion of MAP-contaminated feces, water, or milk. MAP shows an evident tropism for the gastrointestinal tract, crosses the intestinal barrier by interacting with M cells and epithelial cells, and can survive within subepithelial macrophages by inhibiting apoptosis, phagosome acidification, and antigen presentation to the immune system (7–9). MAP-infected animals can progress to the subclinical phase that is characterized by the inhibition of the Th1 pro-inflammatory immune response and MAP persistence within macrophages (10, 11). As the infection progresses, granulomas form, and a host Th1 response is induced, causing intestinal mucosa damage (12, 13). In advanced clinical stages of the disease, a Th2 humoral response emerges, indicating that MAP bacilli escaped granulomas and spread to other tissues and organs (14). MAP control strategies primarily involve identifying and culling infected animals. Currently used diagnostic tests include ELISA for detecting anti-MAP antibodies and real-time qPCR for detecting MAP DNA in fecal samples. While serum ELISA is a simple, fast, and cost-effective diagnostic method, it has low sensitivity in detecting subclinical animals (15). Detecting subclinical infections remains a challenge, and novel diagnostic tools are needed to identify MAP-infected animals in the early and subclinical stages of the infection.

LncRNAs are a type of non-coding RNA (ncRNA) with a length >200 nt that can be coded almost anywhere in the genome, within intergenic regions, within protein-coding genes but on the opposite strand, and within introns and pseudogenes (16). LncRNAs share many characteristics with mRNAs, are transcribed by RNA polymerase II, can be alternatively spliced, are either single-exonic or multi-exonic, are differentially expressed (DE), and are usually polyadenylated at their 3′ ends (17). LncRNAs are relatively shorter than protein-coding genes, exhibit lower expression levels, have fewer but longer exons, have a higher degree of tissue specificity, and have lower levels of homology across species (18, 19). However, lncRNAs from related species may have well-conserved secondary or tertiary structures in structural motifs. Consequently, lncRNA-related gene regulatory roles may be preserved despite a lack of primary sequence conservation (20). The cellular expression levels of lncRNAs are highly correlated with the expression of adjacent protein-coding genes (21). LncRNAs participate in many important regulatory processes such as X chromosome silencing, genome imprinting, chromatic modification, DNA methylation, histone modification, transcription activation, and inhibition, and are useful biomarkers for diagnosis and treatment in various diseases (22–24).

Identification and annotation of novel lncRNAs in the transcriptome of bovine tissues including lung, liver, kidney, white blood cells, mammary gland tissue, and milk somatic cells were previously performed (23, 25–29). However, lncRNAs remain poorly identified and annotated in the bovine genome in comparison to other species such as humans and mice (30). In addition, little is known about the diverse functions of bovine lncRNAs and how they can ultimately impact complex phenotypes including disease outcomes. In MAP-infected macrophages, previous studies have identified novel lncRNAs potentially associated with the regulation of several immune responses including neutrophil degranulation and activation, RNA polymerase function, NF-ƙß signaling pathway, chemokine signaling pathway, cytokine–cytokine receptor interaction, and NOD-like and toll-like receptor signaling pathways (31–33). However, the identification of lncRNAs in the ileocecal valve (ICV) from cows in different stages of the disease has not been addressed before. The objectives of this study were to (1) identify previously annotated and novel lncRNAs present in the bovine ICV transcriptome of 14 Holstein cattle with distinct histopathological lesions in gut tissues and without lesions (controls) using RNA-Seq; (2) identify lncRNAs that are DE between cows with distinct PTB-associated lesions *versus (vs)* controls; (3) predict the target genes of the identified lncRNAs using mRNA expression data from the same animals; and (4) perform quantitative trait locus (QTL) annotation and QTL enrichment analysis using the genomic regions where the DE lncRNAs were located to find additional evidence of their involvement in the regulation of the host immune response against MAP infection. The goal was to provide novel insights into lncRNA regulatory functions in gut tissues of MAP-infected cattle, investigate whether differences in lncRNA profiles reflected in the mRNA data, and explore how they might explain the different disease outcomes.

## 2 Materials and methods

### 2.1 Ethical statement

All methods were conducted in accordance with relevant guidelines and regulations. The study is reported according to the ARRIVE guidelines. The Animal Ethics Committee of the Servicio Regional de Investigación y Desarrollo Agroalimentario (SERIDA) approved the procedures for the animals included in this study. All procedures were authorized by the Regional Consejería de Agroganadería y Recursos Autóctonos of the Principality of Asturias (approval codes PROAE 29/2015 and PROAE 66/2019) and were carried out in compliance with the European Guidelines for the Care and Use of Animals for Research Purposes (2012/63/EU). The cows from which the samples were taken were euthanized for a reason other than collecting samples. The samples were collected by trained personnel following good veterinary practices.

### 2.2 RNA extraction, RNA-Seq library preparation, sequencing, and differential expression analysis

RNA-Seq data used in this study (Gene Expression Omnibus public repository, GEO Accession: GSE137395) were obtained from 14 Holstein cows from a single commercial dairy farm in Asturias, Spain. The cows were classified into three groups based on histopathological analysis: 4 animals without lesions (controls), 5 with focal lesions, and 5 with diffuse lesions, as previously described (34, 35). Total RNA isolation, RNA-Seq library preparation, and sequencing were previously performed (34). In brief, ICV samples were collected at the time of slaughter, stored in RNAlater (Sigma, St Louis, MP), and processed using the RNeasy Mini Kit, according to the manufacturer’s instructions (Qiagen, Hilden, Germany). RNA-Seq libraries were generated using the Illumina NEBNext® Ultra Directional RNA Library preparation kit (Illumina Inc., CA, USA) and were single-end sequenced (1 × 75) using an Illumina NextSeq500 sequencer.

### 2.3 RNA-Seq read trimming, quality control, and transcriptome assembly

**Figure 1:**
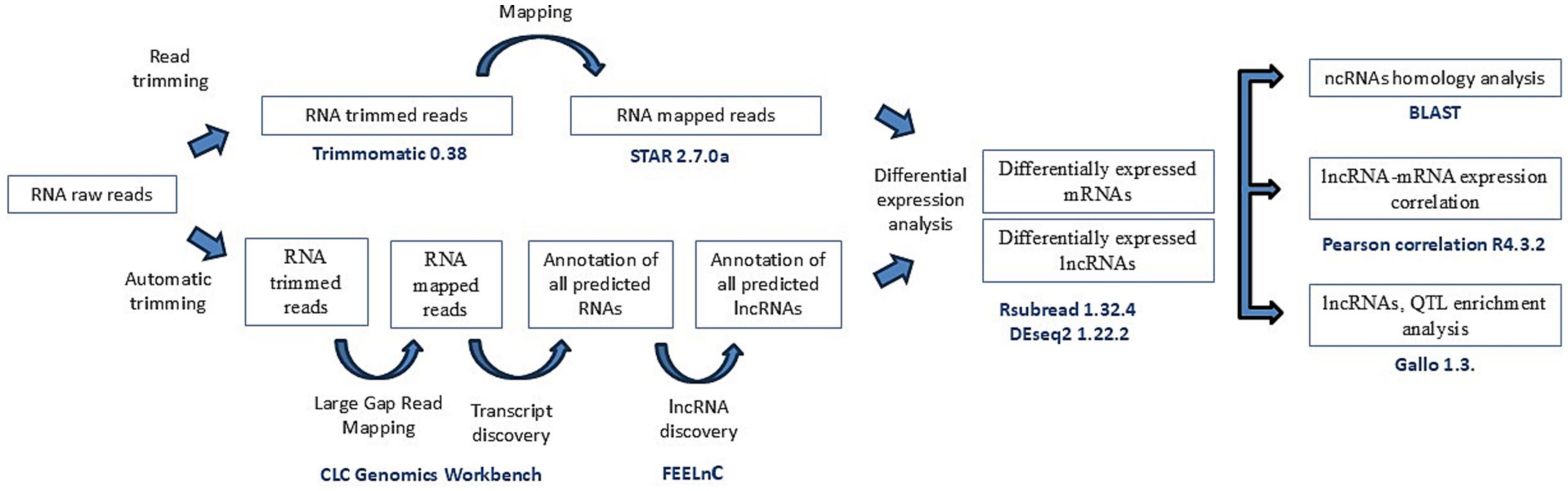
Workflow of the filtering pipeline used in this study. Study workflow from raw fastq reads to lnRNAs identification and differential expression of lncRNAs and nearest genes.

To distinguish lncRNA transcripts from unannotated genes and protein-coding genes, a computational pipeline adapted from a previous study was implemented (25). The pipeline is shown in Figure 1. In brief, raw reads were uploaded to CLC Genomics Workbench (CLC Bio, Aarhus, Denmark) and were trimmed using the automatic trimmer function with a quality trimming score of 0.05. After the reads were trimmed, quality control was performed using the NGS quality control tool of CLC Genomics Workbench, as described by Cánovas et al. (36). The trimmed reads were mapped to the bovine reference genome ARS_UCD1.2.109 using the Large Gap Read Mapping (LGRM) tool in CLC Genomics Workbench, with a length fraction and similarity of 0.7 to exclude paralogous sequence variants and with a minimum contig length of 200 bp, with the following settings: match score: 1, mismatch cost: 2, deletion cost: 3, and insertion cost: 3. The LGRM tool maps reads that span introns without requiring prior transcript annotation, allowing large gaps in-between (37). First, the tool maps the first region of a read with the best match. Second, if 15 bp or more are still unmapped, it returns to the first step and tries to map the remaining sequence. This process continues until the sequence is too short or until the software cannot map it.

### 2.4 Novel and annotated lncRNA identification

The LGRM assembly generated by CLC Genomics Workbench and the bovine reference genome ARS_UCD1.2.109 were used to perform transcript discovery with the following settings: gene merging distance = 50; minimum reads in gene = 10; and minimum predicted gene length = 200 bp. The resulting gtf file containing all the predicted RNAs was used as input of the FlExible Extraction of LncRNA (FEELnc) pipeline to identify lncRNAs. The pipeline generated a new gtf file containing all the identified lncRNAs, which was combined with the gtf of the annotated reference genome.

### 2.5 RNA-Seq alignment and read count

Quality control of the merged file from FeelNC and the annotated gtf reference file was performed using *FastQC 0.12* to identify sequencing read artifacts including sites with low-quality reads (Phred score < 30), duplicated reads, uncalled bases, and potentially contaminated reads (36, 37). Next, the reads were trimmed to remove Illumina adapters and low-quality bases at the start and end of each read using Trimmomatic 0.38 (38). In addition, reads with an average quality score below 20, within a sliding window of 5 nucleotides and with a length <75 nucleotides, were removed. Trimmed reads were mapped to the ARS-UCD1.2.109 reference genome using the Spliced Transcripts Alignment to a Reference aligner *STAR 2.7.0a* (39) with default settings. Read counts were extracted from the alignment files using the function “feature counts” of the *R* package *Rsubread 1.32.4* (40) with the gtf file obtained in the FEELnc pipeline.

### 2.6 Differential expression analysis

Differential expression analysis of all transcripts was performed between all groups (cows with focal lesions *vs.* controls, with diffuse lesions *vs.* controls, and with diffuse *vs.* focal lesions) using the R package *DESeq2 1.22.2* (41). Transcripts were considered as DE between groups when the false discovery rate (FDR) was lower than 0.05. Only those transcripts identified as lncRNAs by the FEELnc pipeline were considered for further analysis. The sequence of each DE lncRNA was extracted from the ARS-UCD1.2.109 reference genome using the coordinates specified in the gtf file. A BLAST analysis was performed to assess if the predicted lncRNAs had homologous sequences with ncRNAs of other species.

### 2.7 Expression of lncRNA-mRNA pairs

LncRNAs tend to exhibit similar expression level patterns to the protein-coding genes that are located nearby (22). To assess if the identified lncRNAs modified the expression of the nearby coding transcript, Pearson’s correlation tests were performed in R using the counts of each lncRNA–mRNA predicted pair, considering a test *p <* 0.05 as significant.

### 2.8 QTL annotation and enrichment analysis

QTL annotation was performed 500 Kbp upstream of the start and 500 Kbp downstream of the end of each DE lncRNA using the R package *Genomic functional Annotation in Livestock for positional candidate LOci* (*GALLO*) (42). In addition, to evaluate which identified QTL was significantly overrepresented in the database, a QTL enrichment analysis using the qtl_enrich() function from *GALLO* was performed. Enriched QTL had an adjusted *p <* 0.05. The genome coordinates of the DE lncRNAs were used, and the QTL gff annotation file was retrieved from release 52 of the Animal QTLdb (43).

## 3 Results

### 3.1 RNA-Seq data and lncRNA identification in ICV samples

**Figure 2:**
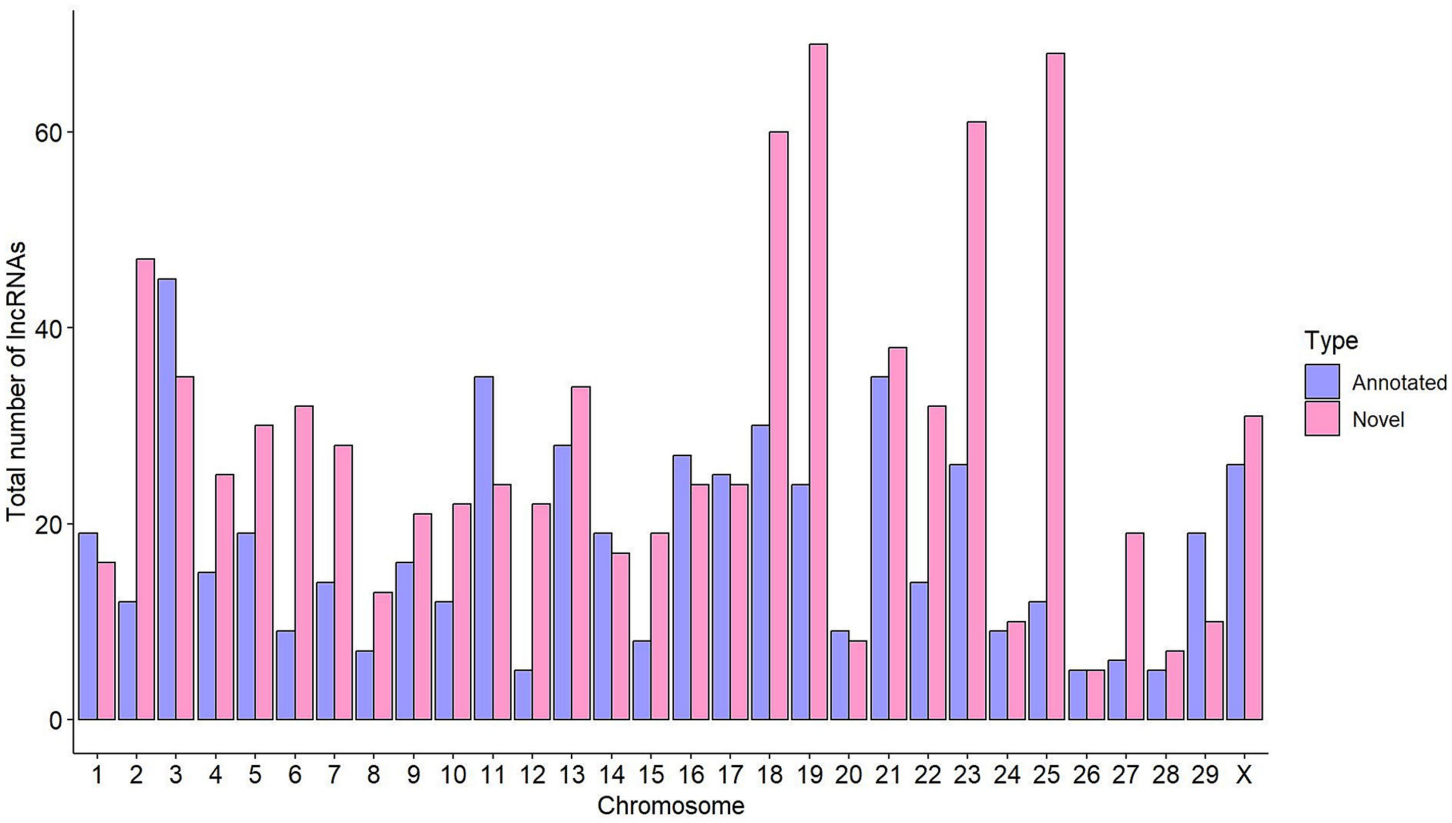
Distribution of lncRNAs across the cow genome. The total number of annotated lncRNAs per chromosome is shown in purple, while the number of novel lncRNAs is indicated in pink.

High-throughput RNA-Seq data derived from ICV samples of 14 animals (4 without lesions, 5 with focal lesions, and 5 with diffuse lesions) were analyzed using the CLC Genomics Workbench (CLC Bio, Aarhus, Denmark), obtaining a mean of 21,579,876 reads per sample. Using LGRM, we observed that a mean of 19,435,468 (90.05%) of these reads mapped to a single location of the bovine reference genome (Supplementary Table 1). A computational pipeline adapted from a previous study (25) was implemented to identify lncRNAs (Figure 1). The FEELnc pipeline identified 1,434 lncRNAs with specificity and sensitivity of 0.962 (Supplementary Figure 1). Figure 2 shows the distribution of the identified lncRNAs across the genome of the cow. Chromosomes BTA19 and BTA18 contained more lncRNAs in comparison to the rest of chromosomes, with each containing 93 and 90 lncRNAs, respectively. Among the 1,434 lncRNAs, 899 (62%) were not annotated in the ARS-UCD1.2.109 bovine reference genome and were considered novel. Interestingly, chromosomes BTA19 and BTA25 account for the highest number of novel lncRNAs; 69 and 68, respectively. Among the 1,434 identified lncRNAs, 1,315 had a predicted gene target. From these 1,315 lncRNAs with a target gene, 600 (46%) were located upstream, 445 (34%) downstream, 195 (15%) in intronic regions, and 75 (5%) in exons.

### 3.2 Differential expression of lncRNAs in ICV from cows with distinct PTB-associated lesions

In total, 1, 6, and 2 lncRNAs were DE in cows with focal lesions *vs.* controls, with diffuse lesions *vs.* controls, and with diffuse *vs.* focal lesions, respectively (Table 1). The DE lncRNAs were located in BTA2, 4, 6, 7, and 12 and mainly upstream of their target mRNA. The longest DE lncRNA (*lnRNA_23401.1*) was located on chromosome BTA4, while the shortest DE lncRNA (*lncRNA_18413.2*) was located on chromosome BTA12. The most highly DE lncRNA, the *lncRNA_18413.2*, was downregulated (log_2_ fold = −5.580) in cows with diffuse lesions compared to control cows.

**Table 1:** Differentially expressed lncRNAs in ICV from Holstein cattle with PTB-associated lesions *vs*. animals without lesions in gut tissues.

Comparison	lncRNA name	Chromosome:start-end (bp)	Size (bp)	Fold change (log2)	*P*-adjusted
Focal vs. no lesions	*lncRNA_1086.1*	2:118402350–118397182	607	−2.251	3.74E-02
Diffuse vs. no lesions	*ENSBTAG00000050406*	7:98006642–98008873	1,212	−1.699	1.43E-02
	*lncRNA_12010.1*	23:25948992–25964039	1,464	−3.149	2.96E-02
	*lncRNA_1086.1*	2:118402350–118397182	607	2.253	1.81E-02
	*lncRNA_18413.2*	12:71169343–71173280	237	−5.58	2.48E-02
	*lncRNA_3598.1 (LOC101907152)*	6:57629779–57631992	429	2.418	4.68E-02
	*lncRNA_2340.1*	4:68890341–68894722	3,805	−1.519	2.28E-02
Diffuse vs. focal lesions	*lncRNA_2340.1*	4:68890341–68894722	3,805	−2.037	1.68E-02
	*lncRNA_14550.3*	NKLS02000910.1:1005548–1016805	2,538	−2.836	3.87E-03

The *lncRNA_1086*.1 was identified in two comparisons: in cows with focal lesions *vs.* controls and with diffuse lesions *vs.* controls. In addition, the *lncRNA_2340.1* was identified in cows with diffuse lesions *vs.* controls and with diffuse *vs.* focal lesions. Only one DE lncRNA (*ENSBTAG00000050406*) was annotated in the ARS-UCD1.2.109 bovine reference genome. BLAST analysis revealed that the *lncRNA_3,598* has been recently annotated in the new version of the bovine reference genome (ARS-UCD1.3) as LOC101907152. BLAST alignment analysis revealed that all the DE lncRNAs shared homology with at least one annotated mammal ncRNA except the *lncRNA_18413.2*. For instance, the *lncRNA_1086.1*, *lncRNA_2340.1*, and *lncRNA_14550.3* shared homology with the human prostate cancer-associated lncRNA (*PRNCR1*), *HOXA-AS3* lncRNA, and the predicted lncRNA *TCONS*-*00241516*, respectively.

### 3.3 Expression of lncRNA–mRNA pairs

Pearson’s correlation tests between lncRNAs and their best-predicted interaction partner revealed that most lncRNAs had significant positive or negative Pearson’s correlation coefficient (*ρ*) with their predicted partners (Table 2). The *lncRNA_2340.1*, lncRNA *ENSBTAG00000050406*, and *lncRNA_12010.1* had a *ρ* of 0.801, 0.905, and 0.686 with their best interaction partners: the *Homeobox A6* (*HOXA6*), the *repulsive Guidance Molecule BMP Co-Receptor B* (*RGMB*), and the transcript *ENSBTAT00000037068*, respectively. The *lncRNA1086.1* had a significant *ρ* of −0.575 with its best interaction partner, the *myotubularin-related protein 9* (*MTMR9*). The *lncRNA_18413.2* and *lncRNA_14550.3* had no significant correlation with their best-predicted interaction partners, and *lncRNA_3598.1* had no predicted interaction partner.

**Table 2:** Pearson’s correlation (*ρ*) tests between lncRNAs and their best-predicted partner.

lncRNA name	Predicted partner gene	Gene fold change (log2)	Pearson’s coefficient (*ρ*)	Correlation (*P*-value)	Location
*lncRNA_1086.1*	*MTMR9*	−0.213	−0.575	3.15E-02	Intronic
*ENSBTAG00000050406*	*RGMB*	−0.611	0.905	8.51E-06	Upstream
*lncRNA_12010.1*	*ENSBTAT00000037068*	−0.011	0.686	6.79E-03	Upstream
*lncRNA_18413.2*	*ENSBTAT00000084572*	0.0522	−0.15	6.06E-01	Upstream
*lncRNA_2340.1*	*HOXA6*	−0.302	0.801	5.70E-04	Upstream
*lncRNA_14550.3*	*ENSBTAT00000078506*	−0.007	−0.057	8.48E-01	Upstream

### 3.4 QTL annotation and enrichment analysis

**Figure 3:**
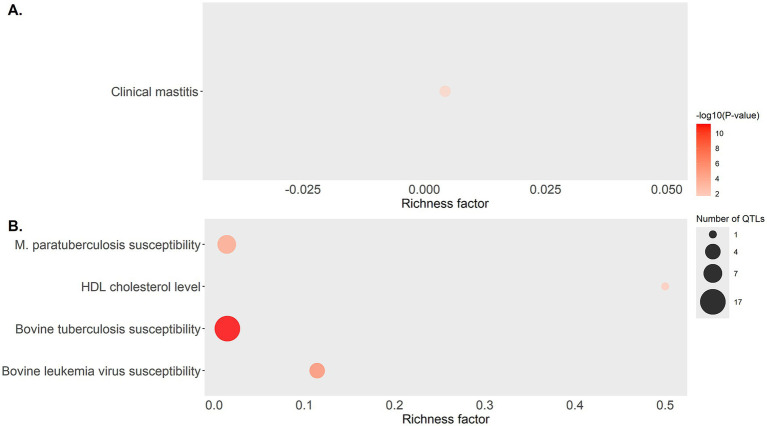
Bubble plot displaying the QTL associated with health traits that were found enriched in the QTL enrichment analysis. QTL enrichment analysis was performed using annotated QTLs located within a 500 Kbp interval of the DE lncRNAs identified in **(A)** cows with focal lesions compared to controls, and **(B)** cows with diffuse lesions compared to controls. The area of the bubbles represents the number of QTL for that QTL class, and the color represents the *p*-value scale (darker color = smaller *p*-value). The richness factor for each QTL represents the ratio of the number of QTL and the expected number of QTL.

QTL annotation 500 Kbp upstream and downstream of the start and end of each DE lncRNA and QTL enrichment analysis were performed using *GALLO*. A total of 37, 123, and 11 QTLs were identified in cows with focal lesions *vs.* controls, with diffuse lesions *vs.* controls, and with diffuse *vs.* focal lesions, respectively (Supplementary Table 2). QTL enrichment analysis was performed to correct the large number of QTL in the database associated with the more studied traits (Figure 3). In cows with focal lesions *vs.* controls, significant enrichment of QTLs previously associated with clinical mastitis was observed. In the comparison of cows with diffuse lesions *vs.* controls, significantly enriched QTLs were associated with HDL cholesterol, bovine leukemia, bovine tuberculosis, and paratuberculosis susceptibility.

## 4 Discussion

LncRNAs participate in host–pathogen interactions that alter the resulting immune response (44), but their functional annotation in cattle is very limited, and their role in the regulation of the host immune response against mycobacterial infections remains unknown. Furthermore, the identification of lncRNAs is challenging due to their lower expression levels than mRNAs. Recent studies have shown that the low expression of lncRNAs can be essential for their functional role by ensuring specificity to their regulated targets (45). A common approach to discover and annotate putative lncRNAs is to consider transcripts that have nucleotide lengths >200 nt and that show little to no evidence of protein-coding potential (26, 30). In this study, RNA-Seq data from ICV samples of 14 Holstein cows without lesions and with two different PTB-associated lesions in gut tissues (focal and diffuse) allowed the identification of 1,434 lncRNAs, 899 of which were novel. The genomic features of the identified lncRNAs were in concordance with earlier observations in cattle and in other animal species. For instance, the identified lncRNAs had lower expression levels than their target mRNAs, and their length was shorter due to the lower number of exons in the transcripts. In our study, the predominant distribution of the identified lncRNAs in chromosomes BTA18, BTA19, and BTA25 was also consistent with the predominant location of the lncRNAs previously identified in MAP-infected macrophages (32, 33). To the best of our knowledge, this is the first study that provides novel insights into the role of lncRNAs in the bovine ICV, the primary site of MAP infection, and their interplay with mRNAs during MAP infection in cattle. Whether the identified lncRNAs are specific to MAP infection and may serve as potential biomarkers to detect infected animals needs to be further investigated.

Eight lncRNAs were DE between cows with different PTB-associated lesions *vs.* controls, five of which were unannotated and could be considered novel (*lncRNA_1086.1*, *lncRNA_12010.1*, *lncRNA_18413.2*, *lncRNA_2340.1*, and *lncRNA_14550.3*). Four of the five novel DE lncRNAs (*lncRNA_1086.1*, *lncRNA_12010.1*, *lncRNA_2340.1*, and *lncRNA_14550.3*) had partial homology with at least one annotated mammal ncRNA. We observed no overlap between the DE lncRNAs and previously published bovine lncRNAs identified in MAP-infected macrophages (31, 33, 46) and jejunum samples from MAP-infected cattle (47). This might be because the bovine lncRNA library is not fully annotated and some lncRNAs show tissue-specific expression patterns (48). The lack of overlap between the DE lncRNAs identified in this study and those reported in previous studies may be due to other factors such as methodological differences, variations in lncRNA discovery algorithms, or differences in study samples. None of the genes close to DE lncRNAs were previously associated with susceptibility to PTB in the literature.

Inferring lncRNA biological roles is challenging, particularly for dairy cattle, where the functional annotation of lncRNAs is limited. We inferred the putative biological roles of the identified lncRNAs by assessing the function of neighboring protein-coding genes and the lncRNAs-mRNA co-expression patterns. In cows with focal lesions *vs.* controls, the *lncRNA1086.1* was downregulated (log_2_ fold = −2.251). In contrast, in cows with diffuse lesions *vs.* controls, the *lncRNA1086.1* was upregulated (log_2_ fold = 2.253). In both comparisons, the *lncRNA1086.1* was predicted to interact with the *MTMR9* gene, and the expression levels of the *lncRNA1086.1* and the *MTMR9* were negatively correlated (*ρ* = −0.575). The *MTMR9* interacts with the *MTMR6*, and this interaction is critical for lipid binding, catalytic activity, and protein stability of *MTMR6*, and the depletion of both proteins promotes apoptosis (49). Therefore, we hypothesize that the decrease in the *lncRNA1086.1* expression observed in the cows with focal lesions *vs.* controls (log_2_ fold = −2.251) would cause an increase in *MTMR9* expression and an inhibition of apoptosis during the subclinical stage of MAP infection, which leads to MAP intracellular survival and persistence without infected macrophages. In contrast, the increase in *lncRNA1086.1* expression (log_2_ fold = 2.253) in cows with diffuse lesions *vs.* controls would have the opposite effect. Therefore, our results suggest that the *lncRNA1086.1* could be modulating apoptosis in distinct stages of MAP infection. Interestingly, the sequence of the *lncRNA_1086.1* shared 91% homology with the *PRNCR1*. This lncRNA has been reported to be an oncogenic transcript participating in the pathogenesis of several kinds of cancers, and some single-nucleotide polymorphisms within this lncRNA affect cancer risk (50, 51). *PRNCR1* levels in tumors were shown to have diagnostic and prognostic importance, and comparatively higher expression levels of this lncRNA were also found in blood exosomes of patients with pancreatic carcinoma, hepatocellular carcinoma, and colorectal cancer than that of normal controls.

In cows with diffuse lesions *vs.* controls, downregulation of the *ENSBTAG00000050406* lncRNA was observed (log_2_ fold = −1.699). The *ENSBTAG00000050406* was predicted to interact with the *RGMB* gene, resulting in a downregulation of the *RGMB* expression (*ρ* = 0.905). The dysregulation of *RGMB* has been implicated in the development and progression of various malignancies in humans, including breast, prostate, lung, and colorectal cancers; melanomas, and osteosarcomas (52, 53). Interestingly, the inhibition of *RGBM* expression in RAW264.7 or J774 macrophages increased interleukin 6 (IL-6) expression, while *RGMB* overexpression inhibited IL-6 expression in a ligand-dependent manner (54). In our study, therefore, the observed decrease in the expression of the lncRNA *ENSBTAG00000050406* (log_2_ fold = −1.699) could cause downregulation of the *RGMB* (log_2_ fold = −0.611) and upregulation of IL-6, thus enhancing the pro-inflammatory immune response. Rapid production of IL-6 contributes to host defense during infection and tissue injury, but excessive IL-6 synthesis is involved in PTB pathology. Other lncRNAs with differing expression levels in the comparisons of cows with diffuse lesions *vs.* controls were the *lncRNA 12010.1* (log_2_ fold = −3.149), *lncRNA 18413.2* (log_2_ fold = −5.580), and *lncRNA 3598.1* (log_2_ fold = 2.418).

In the cows with diffuse lesions, downregulation of the *lncRNA_2340.1* was observed. The *lncRNA_2340.1* was predicted to interact and downregulate *HOXA6* gene expression (*ρ* = 0.801). The homeobox genes (*HOX*) are a group of important transcriptional regulatory factors that act on target genes and are highly conserved in evolution (55–57). They can participate in embryonic development, cell identification, cell differentiation, cell metabolism, apoptosis, autophagy, and other processes. In gastric cancer and colorectal cancer, *HOXA6* can inhibit the apoptosis of tumor cells by binding to other genes or acting on other pathways to promote the occurrence and progression of tumors (58, 59). Taking these results into account, we suggest an important immunomodulatory role of the *lncRNA_2340.1* in MAP-infected cows with diffuse lesions by suppressing *HOXA6* expression and enhancing apoptosis in the clinical state of MAP infection. Interestingly, the *lncRNA_2340.1* shared 84.7% homology with the human *HOXA-AS3* lncRNA. Dysregulation of the *HOXA-AS3* lncRNA contributes to the development of multiple cancer types (60). Finally, in the comparison of cows with diffuse *vs.* focal lesions, the *lncRNA_145550.3* was downregulated (log_2_ fold = −2.836), and although it was predicted to interact with the *ENSBTAT00000078506*, the interaction was not statistically significant (*p*-value = 0.848). Interestingly, we found that the *lncRNA_145550.3* shared 81.6% of homology with the human lncRNA *TCONS-00241516.*

The enrichment analysis of QTLs revealed that the DE lncRNAs identified in cows with focal lesions *vs.* controls were located in two QTLs previously associated with clinical mastitis (p-value 0.045). Previous studies found that the incidence of mastitis, PTB, and the coexistence of both infections was significant (61). Therefore, these QTLs could make the cows more susceptible to clinical mastitis and PTB. In the cows with diffuse lesions *vs.* controls, the DE lncRNAs overlapped with QTL previously associated with HDL cholesterol levels, bovine tuberculosis, bovine leukemia virus, and MAP susceptibility. If the identified DE lncRNAs have a fundamental role in regulation due to their interactions with transcripts nearby, this might provide key insights for the development of breeding programs for PTB resistance.

## 5 Conclusion

Using RNA-Seq data from ICV, the primary site of MAP infection, a total of 1,434 lncRNAs were identified, 899 of them were novel. Among the lncRNAs dysregulated in cattle with PTB-associated lesions, seven were linked to genes DE in ICV such as *MTMR9*, *RGMB*, *HOXA6, ENSBTAT00000037068*, *ENSBTAT00000084572*, and *ENSBTAT00000078506*. While some DE lncRNAs (*ENSBTAG00000050406* and *lncRNA_2340.1*) activate genes with roles in apoptosis, host immune response, and inflammation, it is also conceivable that MAP utilizes some host lncRNAs (*lncRNA1086.1*) to block the induction of anti-bacterial genes and apoptosis. Interestingly, two of the DE lncRNAs (*lncRNA_1086.1* and *lncRNA_2340.1*) were homologous with the human *PRNCR1* and *HOXA-AS3* lncRNAs that act as oncogenes or tumor suppressors, respectively. The identified lncRNAs were located in regions previously associated with MAP susceptibility and other bovine diseases such as clinical mastitis, bovine tuberculosis, and bovine leukemia virus, suggesting that the identified lncRNAs might have a conserved function in response to MAP infection and potentially to other pathogens as well. Future research should focus on elucidating the specific mechanism by which the identified lncRNAs regulate gene expression and disease pathogenesis.

## Data Availability

The datasets presented in this study are deposited in the Gene Expression Omnibus repository, accession number GSE137395.

## Supplementary material

SUPPLEMENTARY FIGURE 1 Two-graph receiver operating characteristic curve (ROC) to determine the optimal coding probability cutoff value. Dashed black lines represent the threshold chosen by FEELnc codpot as the point that maximizes sensitivity (blue lines) and specificity (red lines) of prediction of lncRNAs.

SUPPLEMENTARY TABLE 1 Summary statistics of the LGRM step.

SUPPLEMENTARY TABLE 2 QTL enrichment analysis.
