# Supplementary figures and images for: Genome-wide long non-coding RNA expression profile and its regulatory role in the ileocecal valve from Mycobacterium avium subsp. paratuberculosis-infected cattle
Source: Front Vet Sci. 2025 Jun 5;12:1601267. doi: 10.3389/fvets.2025.1601267 (PMC12176553; doi:10.3389/fvets.2025.1601267)

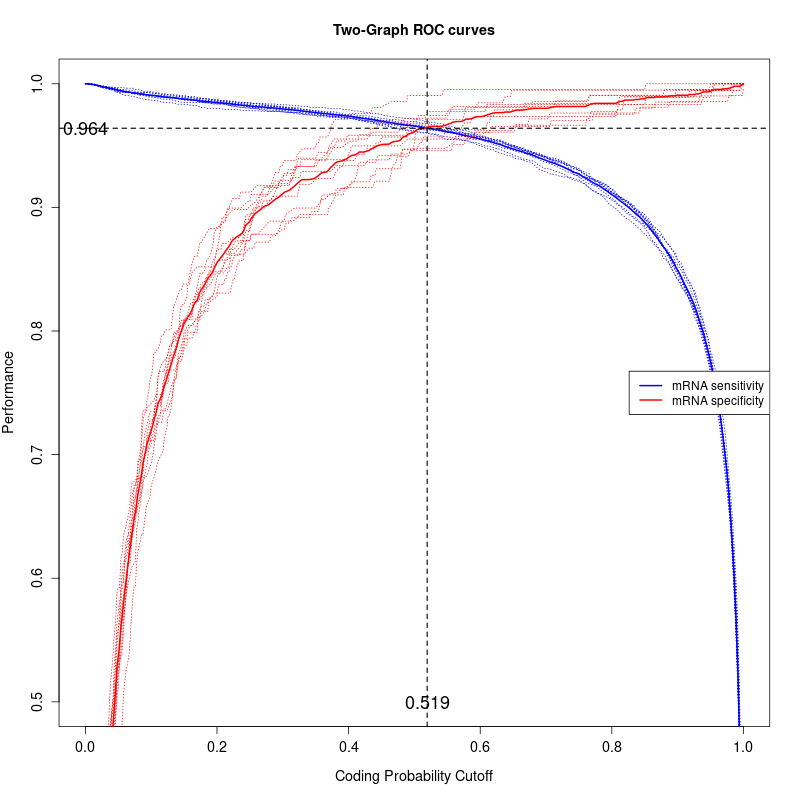

Supplement: SUPPLEMENTARY FIGURE 1 — Two-graph receiver operating characteristic curve (ROC) to determine the optimal coding probability cutoff value. Dashed black lines represent the threshold chosen by FEELnc codpot as the point that maximizes sensitivity (blue lines) and specificity (red lines) of prediction of lncRNAs. [file Image_1.tiff]
