# Supplementary material for: Genome-wide long non-coding RNA expression profile and its regulatory role in the ileocecal valve from Mycobacterium avium subsp. paratuberculosis-infected cattle
Source: Front Vet Sci. 2025 Jun 5;12:1601267. doi: 10.3389/fvets.2025.1601267 (PMC12176553; doi:10.3389/fvets.2025.1601267)
Supplement: SUPPLEMENTARY TABLE 1 — Summary statistics of the LGRM step. [file Table_1.docx]

**Supplementary Table 1. Summary statistics of the LGRM step.**

| ID | Histopathological analysis | N° of reads | Uniquely mapped | % | Non-specifically mapped | % |
| --- | --- | --- | --- | --- | --- | --- |
| 1 | Negative | 22,040,380 | 20,084,354 | 91.13% | 1,956,026 | 8.87% |
| 2 |  | 21,761,682 | 19,825,896 | 91.10% | 1,935,786 | 8.90% |
| 3 |  | 22,040,677 | 19,969,566 | 90.60% | 2,071,111 | 9.40% |
| 15 |  | 28,992,334 | 25,899,734 | 89.33% | 3,092,600 | 10.67% |
| 4 | Focal | 21,542,745 | 19,760,925 | 91.73% | 1,781,820 | 8.27% |
| 5 |  | 22,587,852 | 20,510,890 | 90.80% | 2,076,962 | 9.20% |
| 6 |  | 18,557,409 | 16,680,123 | 89.88% | 1,877,286 | 10.12% |
| 7 |  | 20,163,548 | 18,296,635 | 90.74% | 1,866,913 | 9.26% |
| 8 |  | 19,902,156 | 18,289,319 | 91.90% | 1,612,837 | 8.10% |
| 10 | Diffuse | 17,870,903 | 15,896,718 | 88.95% | 1,820,294 | 10.19% |
| 11 |  | 18,725,483 | 16,080,218 | 85.87% | 1,890,240 | 10.09% |
| 12 |  | 25,959,638 | 23,078,377 | 88.90% | 2,881,261 | 11.10% |
| 13 |  | 17,646,572 | 15,828,585 | 89.70% | 1,817,987 | 10.30% |
| 14 |  | 24,326,881 | 21,895,209 | 90.00% | 2,431,672 | 10.00% |
